# Length of Time to Clinical Improvement After Orthopedic Oncology Surgery in Patients With Metastatic Cancer: A Multi‐Institution Patient‐Reported Outcome Study

**DOI:** 10.1002/jso.27932

**Published:** 2024-11-28

**Authors:** John Groundland, Jacqueline Hart Tokson, Anne Hakim, Amy Cizik, Alan Blank, Daniel Lerman, Kevin Jones, R. Lor Randall

**Affiliations:** ^1^ Department of Orthopedic Surgery University of Utah Salt Lake City Utah USA; ^2^ Department of Orthopedic Surgery Rush University Chicago Illinois USA; ^3^ Department of Orthopedic Surgery Colorado Limb Consultants Denver Colorado USA; ^4^ Department of Orthopedic Surgery University of California Davis California USA

**Keywords:** metastatic bone lesion, orthopedic oncology, pathologic fracture, patient‐reported outcome

## Abstract

**Background:**

Currently, there is a paucity of data that describes the length of time required to realize improvement in pain and function following surgery for patients with metastatic cancer to bone.

**Methods:**

One hundred patients with impending or completed pathologic fractures due to metastatic cancer to bone were enrolled in this prospective cohort study. Outcomes were measured with a Computer Adaptive Test of Patient Reported Outcomes for Pain Interference and Physical Function domains, to determine the time required to achieve a Minimal Clinically Important Difference (MCID) in the tested domains.

**Results:**

Eighty‐one patients were included in the analysis. Thirty‐two patients (39.5%) survived and completed the follow‐up to 1 year, while 23 (28.4%) died before the end of the data collection. Fifty‐one patients (63.0%) achieved at least a 5‐point improvement in Physical Function and 59 (72.8%) achieved at least a 5‐point improvement in Pain Interference. The time to achieve the MCID was 6 weeks for the Physical Function and 4 weeks for the Pain Interference domain.

**Conclusion:**

The majority of patients with impending or completed pathologic fractures due to metastatic cancer see clinically important improvements in pain and function after surgery in an average of 4 and 6 weeks, respectively.

## 1 Background

When cancers metastasize to bone, the biology of the cancer, if left unchecked, may lead to progressive osteolysis and weakening of the bone. With decreasing structural integrity of the bone, the natural history of this process leads to a period of time when the bone is at a high risk for fracture. Unabated, an impending fracture can convert to a realized fracture, potentially leaving a patient who has an advancing cancer with severe pain and profound dysfunction [1]. It is for this reason that orthopedic oncologists try to identify cancer patients who are at risk for a pathologic fracture and provide prophylactic fixation when indicated. The medical literature supports this practice [2, 3, 4, 5, 6, 7, 8, 9]. However, there are many cancer patients who are deemed to be at high risk for a pathological fracture, as well as patients who have sustained a completed fracture and who may be facing a very limited life expectancy. One may rightly question the utility of spending weeks or months recovering from orthopedic surgery when those weeks or months may be all of the remaining time of life they have. Currently, there is a paucity of data published in the medical literature that describes the length of time it takes to realize improvement in pain and function following surgery for patients with metastatic cancer to the bone with impending or realized pathologic fractures. Such information could provide patients and caregivers with insight into the relative benefits and limitations of adding surgical intervention to the multidisciplinary care of patients with metastatic cancers to bone.

Measurement of patient improvement and satisfaction with orthopedic care has evolved over the past several decades. Many of the early clinical tools were physician‐based or physician‐administered [10, 11, 12, 13]. Such tools may have led to bias or distortion of measurable outcomes. To combat this potential bias, there has been growing use and acceptance of Patient Reported Outcome Measures (PROMs), which rely on direct patient responses to targeted questions, typically administered in a short, computer‐adaptive session [12, 14, 15, 16, 17]. These questionnaires are concise and demonstrate satisfactory validity to a wide array of patients and clinical circumstances [18, 19, 20, 21, 22, 23, 24].

The purpose of this study is to investigate the time required for patients to report improvements in pain and physical function following orthopedic surgery for impending or realized pathologic fracture due to metastatic cancer to bone. We utilized the Pain Interference and Physical Function tests from the Patient‐Reported Outcomes Measurement Information System Computer Adaptive Test (PROMIS CAT, HealthMeasures) [25, 26, 27], administered at standardized time points over the course of 1 year to determine the pain and functional recovery trends demonstrated in this orthopedic oncology patient population.

## 2 Materials and Methods

### 2.1 Study Design

This was a prospective, non‐randomized, non‐blinded cohort study performed at three institutions across the United States. Before data collection, Institutional Review Board approval was obtained. The enrollment goal was 100 patients who were deemed to have an impending fracture or a completed fracture due to metastatic cancer to bone. Inclusion criteria consisted of impending or completed pathologic fracture of a bone, as determined by an orthopedic surgeon with fellowship training in musculoskeletal oncology; orthopedic surgical intervention by an orthopedic oncologist to fix the impending or realized fracture; pathology‐proven metastatic cancer to the bone in question; patients at least 30 years of age; English language proficiency; and cognitive proficiency to complete the computer‐adaptive test. Exclusion criteria included: non‐oncologic diagnoses; no deemed risk for impending or realized fracture; patients under 30 years of age; lack of English language proficiency; patients with insufficient ability to independently complete the computer‐adaptive test.

Demographic and background data for each indicated and consented patient were entered into a protected data repository, the Research Electronic Data Capture (REDCap) system. These data included age, gender, self‐reported ethnicity, body‐mass index, cancer diagnosis, neoadjuvant treatments such as chemotherapy and radiation, impending or realized fracture, the bone involved, preoperative function by Karnofsky score [28] and Mirel criteria [29], and surgery performed. The type of surgery performed was left to the discretion of the treating surgeon and the patient, based on the evaluated needs of the patient and an individualized preoperative informed consent discussion. Surgeries included intramedullary rod fixation, hemiarthroplasty, total joint arthroplasty, and plate and screw fixation. No patient was randomized to a treatment modality. Those who did not consent to surgery were not included in the study, nor were they followed for comparison's sake.

### 2.2 Measures

The Pain Interference and Physical Function domains of the PROMIS CAT from HealthMeasures were utilized to determine each patient's pain and level of function. The computer adaptive tests were administered on computer tablets (iPad, Apple, Cupertino, CA) with an anticipated average time to completion of approximately 1 min. The tests were administered at set time periods, relative to the surgical intervention. Twelve tests were planned for each patient: an immediate preoperative (baseline) test, followed by postoperative assessments on Day 1, Week 2, Week 4, Week 6, Week 10, Month 3, Month 4, Month 5, Month 6, Month 9, and Month 12. The responses were collected and entered into the REDCap system, matching the demographic and background data for each patient.

Results from each computer adaptive test were converted to *T*‐scores, through the HealthMeasures website and data systems [25, 26, 27]. This produces an individual *T*‐score for each patient's test, as compared to the historical response of the general population in the United States to the same test, where the US population's score is standardized to 50 with a standard deviation of 10. For the Physical Function domain of the test, a lower score indicates the worse function, whereas, for the Pain Interference domain, a higher score indicates a greater amount of pain experienced by the patient. Thus, clinical improvement is seen with a rising Physical Function domain score and a dropping Pain Interference score. As both domains approach 50, the tested cohort is assumed to be approximating the level of function or pain of the general population.

We used the data sets from HealthMeasures as well as the available medical literature to set the Minimal Clinically Important Difference (MCID). In regard to PROMs, the MCID is a concept that explores the question of how much change in a patient's *T*‐score for a certain tested domain reflects a meaningful change in that patient's life. There is no single, universal number that defines MCID; rather there is reason to believe that *T*‐score MCIDs vary by domain and by disease process. Based on a search of the literature, we set the MCID for the Physical Function domain as a change in T‐score of +5.0 and the MCID for Pain Interference at −5.0 and −7.5 [18, 19, 23, 24]. The −7.5 value for the Pain Interference domain was added to the more commonly accepted 5.0 change due to the published data by Bongers et al. [19]. In this study, the authors specifically studied patients with skeletal metastasis to the lower extremities with impending pathologic fracture, and they determined that a −7.5 point change in Pain Interference was appropriate for this specific patient population. As this was just one study, however, we included the more traditional −5.0 point change for Pain Interference as another point of data.

### 2.3 Statistics

Descriptive statistics for the demographic and treatment data were determined and tabulated. PROMIS scores were normalized as *T*‐scores and standard deviations were calculated, with a score of 50 and a standard deviation of 10 representing the average score and variation of the general population in the United States. Statistical tests to determine any potential differences between the treatment groups (type of surgical intervention given) with regard to their *T*‐scores were not done, as the purpose of the study was to describe the time to improvement in pain and function for the entire cohort, not to compare the various treatment groups.

## 3 Results

Of the 100 patients who enrolled in the study, 81 were included in the final analysis. Nineteen patients were excluded, due to the following reasons: 12 for not completing the baseline survey and 7 for lack of any meaningful demographic or treatment data. Table 1 lists the demographic data of the patients. The average age of the patients was 61.8 years, with a range of 33−92 years. The majority of the patients were female (*n* = 47, 58%) and self‐identified as non‐Hispanic White (*n* = 63, 77.8%). The primary cancer diagnoses included several subtypes of carcinoma, as well as melanoma, multiple myeloma, and two metastatic sarcomas. Breast cancer was the most common diagnosis, occurring in 19 patients, representing 23.5% of the 81 subjects in the study.

**Table 1:** Patient demographics and diagnoses—Entire cohort of patients with impending or completed pathologic fracture due to metastatic cancer to bone.

		*n*	
All patients		81	
Age^a^		79	61.8 years (33−92)
Gender	Female	47	58.0%
	Male	34	42.0%
Race/ethnicity	White	63	77.8%
	Hispanic	4	4.9%
	Native American	1	1.2%
	Not reported	13	16.0%
BMI^a^		73	28.9 kg/m^2^ (18.2−52.6)
Diagnosis^b^	Breast	19	23.5%
	Prostate	9	11.1%
	Renal	10	12.3%
	Lung	2	2.5%
	Gastrointestinal	3	3.7%
	Melanoma	5	6.2%
	Multiple myeloma	15	18.5%
	Other	18	22.2%

a Patient age and body mass index (BMI) are reported as the mean, with the range in parentheses.

b Diagnosis: Diagnoses are listed by the site of origin of the metastatic cancer.

Table 2 lists preoperative and operative data for the cohort. The majority of the patients underwent surgery to the femur (*n* = 59, 72.8%), with the acetabulum as the second most common anatomical site (*n* = 14, 17.3%). The average Karnofsky physical performance score was 61.1, representing a patient who “requires occasional assistance, but is able to care for most personal needs.” The range was from a low of 20 (“very sick with active supportive treatment necessary”) to a high of 100 (normal) [28]. Mirel score, a method of assessing the risk of pathologic fracture based on radiographic and clinical assessment, was assessed in all patients that did not have a completed fracture (*n* = 58) [29]. The average score was 10.3, with a range of 8−12. Fifteen patients (18.5%) had no other known sites of metastatic disease, while 58 patients (71.6%) had known additional sites of metastatic disease; 8 patients did not have this data recorded in the database. For neoadjuvant treatments, the majority had chemotherapy (*n* = 49, 60.5%), while 31 patients (38.3%) had preoperative radiation; 21 patients (25.9%) received bone‐building medications before surgery, in the form of bisphosphonates or denosumab. Intramedullary rod fixation was the most common surgical procedure performed, with 46 patients (56.8%) receiving this intervention, followed by arthroplasty (total or hemi) (*n* = 22, 27.2%); the record for 6 patients was incomplete for surgical intervention, thus leaving them “unreported.” Of note, 13 of the 22 arthroplasties were done for realized fractures, while 4 realized fractures were treated with intramedullary rods and 2 were treated with plate and screws; 2 of the cases of completed fracture did not report the surgical intervention.

**Table 2:** Preoperative and operative data—Entire cohort of patients with impending or completed pathologic fracture due to metastatic cancer to bone.

		*n*	
Anatomic site of surgery	Acetabulum	14	17.3%
	Femur	59	72.8%
	Tibia	1	1.2%
	Humerus	7	8.6%
Karnofsky score^a^		71	61.1 (20–100)
Mirel score^a^		54	10.3 (8–12)
Preoperative pain	Mild	6	7.4%
	Moderate	6	7.4%
	Severe/functional	69	85.2%
Preoperative fracture	Realized/completed	23	28.4%
	Impending	58	71.6%
Additional sites of metastasis	None	15	18.5%
	Osseous	36	44.4%
	Visceral + osseous	22	27.2%
	Unreported	8	9.9%
Neoadjuvant treatments	Chemotherapy	49	60.5%
	Radiation	31	38.3%
	Bisphosphonate	15	18.5%
	Denosumab	6	7.4%
	None	22	27.2%
	Unreported	4	4.9%
Type of surgery	Intramedullary rod	46	56.8%
	Plate and screw	7	8.6%
	Arthroplasty	22	27.2%
	Unreported	6	7.4%

a Karnofsky score [28] and Mirel score [29] are reported as the mean with the range in parentheses.

Table 3 lists the postoperative data, including follow‐up. Thirty‐two patients (39.5%) completed the full year of the study, with 19 of those completing all 12 surveys. Of those that did not complete the full year of the study, 23 (28.4%) died before the year was done, 8 (9.9%) dropped out due to ill health, and 18 (22.2%) were lost to final follow‐up. For those that died, the average time to death was 4 months (range 1−11 months), and their average number of surveys completed was 4.2 (range 2−9). The average number of surveys completed by those that did not die during the study period was 8.2 (range 3−12). The average number of surveys completed by the entire cohort was 7.0, with a range of 2−12 surveys completed.

**Table 3:** Postoperative data—Entire cohort of patients with impending or completed pathologic fracture due to metastatic cancer to bone.

		*n*	%
Follow‐up	Survived and completed follow‐up to 1 year	32	39.5
	Died before 1 year	23	28.4
	Dropped out of study due to Ill health	8	9.9
	Lost to follow‐up during the year	18	22.2
Number of surveys completed	All 12	19	23.6
	6−11	33	40.7
	3−5	29	35.8
Achieved at least a 5‐point improvement in physical function^a^	Yes	51	63.0
	No	30	37.0
Achieved at least a 5‐point improvement in pain interference^a^	Yes	59	72.8
	No	22	27.2
Achieved at least a 7.5‐point improvement in pain interference^a^	Yes	52	64.2
	No	29	35.8

a Patient Reported Outcome Measures Computer Adaptive Test, HealthMeasures: www.healthmeasures.net/promis.

**Figure 1:**
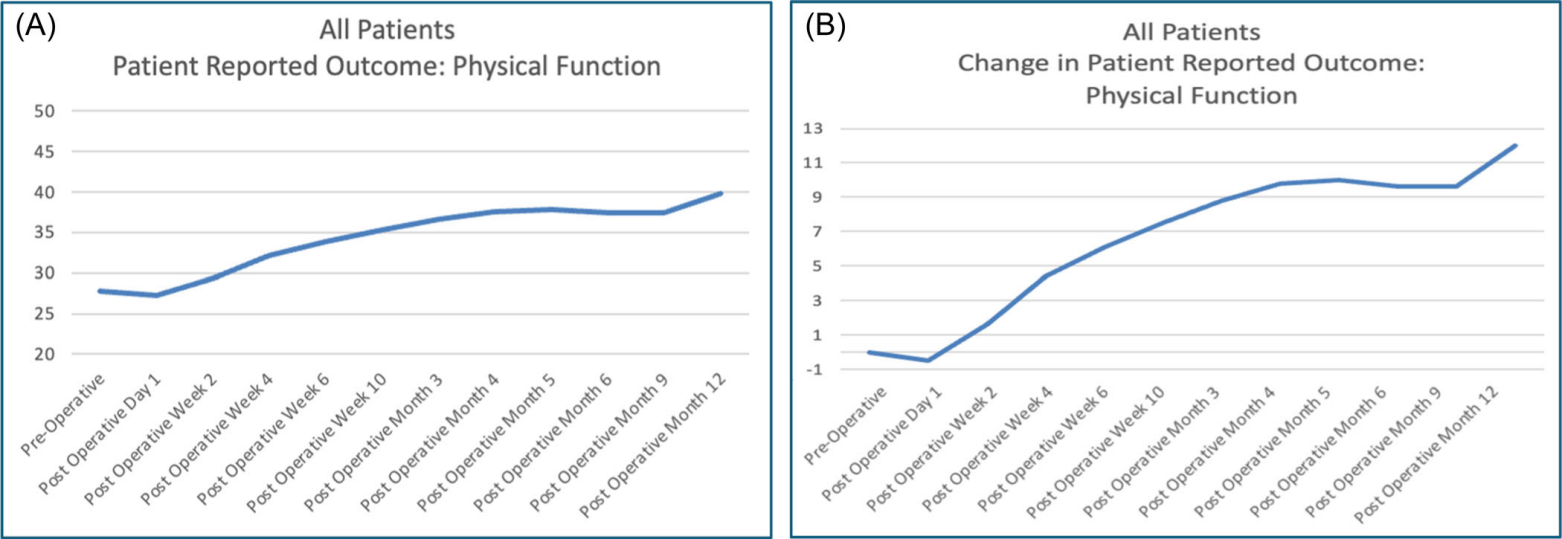
Mean Physical Function domain T‐scores for patients with impending or realized pathologic fracture due to metastatic cancer to bone, before and after orthopedic intervention. (A) Average *T*‐score for the entire cohort over time, where a score of 50 represents historical control of the average US population (standard deviation ±10), and a lower score reflects worse function. (B) Change in the average *T*‐score of the studied cohort relative to their test taken at baseline, immediately before surgery, with a change in score of +5.0 set as the Minimally Clinically Important Difference.

Tables 3 and 4 show the results of the Physical Function domain of the PROMIS CAT taken by the patients. The average *T*‐score at the time of their preoperative baseline assessment was 27.8 (standard deviation 7.44, standard error 0.83). For this cohort, 63% (*n* = 51) achieved at least a 5‐point improvement in their Physical Function score following surgical intervention. The average time to achieve this level of change for the entire cohort was 6 weeks after surgery. These changes in the T‐score were durable, with no regression in function during the study period for the cohort as a whole. Additional improvements in the Physical Function scores were noted after this 6‐week mark (Table 4, Figure 1), with another 5‐point gain in the Physical Function *T*‐score noted by the end of the study period. However, a subset of patients, 18.5% (*n* = 15), experienced a decrement in function, as demonstrated by scores that declined after surgery and remained worse than baseline during follow‐up.

**Table 4:** Physical function domain: Average T‐scores for patients undergoing orthopedic intervention for impending and realized pathologic fractures due to metastatic cancer to bone, where a score of 50 with a standard deviation of 10 is standardized to the average US population.

	All patients *T*‐score mean ± SD (*n*)	Arthroplasty *T*‐score mean ± SD (*n*)	Plate/screw *T*‐score mean ± SD (*n*)	IM rod *T*‐score mean ± SD (*n*)
Preoperative	27.8 ± 7.4 (81)	27.1 ± 7.1 (22)	29 ± 10.6 (7)	28.3 ± 7.5 (46)
Postoperative Day 1	27.3 ± 7.9 (65)	29 ± 8.9 (19)	30.9 ± 5.2 (6)	26.4 ± 7.9 (33)
Postoperative Week 2	29.4 ± 7.9 (66)	32.3 ± 8.3 (20)^a^	26.7 ± 5.8 (6)	28.4 ± 8.1 (33)
Postoperative Week 4	32.2 ± 8.3 (45)	31.2 ± 6.6 (14)	28.7 ± 8.6 (7)	33.3 ± 9.4 (24)^a^
Postoperative Week 6	33.9 ± 8.1 (50)^a^	36.9 ± 7.3 (14)	30.9 ± 5.3 (5)	33.1 ± 8.7 (30)
Postoperative Week 10	35.3 ± 9.4 (46)	37.6 ± 9.4 (16)	29.6 ± 10.0 (4)	34.7 ± 9.4 (25)
Postoperative Month 3	36.6 ± 8.3 (39)	33.6 ± 7.7 (12)	41.4 ± 9.9 (3)^a^	37.8 ± 8.3 (23)
Postoperative Month 4	37.6 ± 7.5 (37)	37.1 ± 7.1 (11)	35.9 ± 1.7 (3)	38 ± 8.3 (23)
Postoperative Month 5	37.8 ± 8.7 (32)	40.8 ± 7.7 (10)	38.3 ± 1.8 (2)	36.5 ± 9.5 (19)
Postoperative Month 6	37.4 ± 8.1 (35)	40.3 ± 6.5 (12)	33.5 ± 0.3 (3)	36.8 ± 9.2 (19)
Postoperative Month 9	37.4 ± 8.8 (30)	43.1 ± 6.9 (9)	35.2 ± 1.5 (3)	35.1 ± 8.5 (17)
Postoperative Month 12	39.8 ± 8.6 (27)	43.8 ± 7.6 (8)	44.8 ± 15.8 (2)	37.3 ± 7.9 (17)

*Note:* Six patients did not have their reconstruction listed in the REDCap database, but were included in the total cohort, as the remainder of their data was complete. Therefore, the listed numbers of each reconstruction may not add to the number listed in the all patients group.

Abbreviation: SD, standard deviation.

a First time each group's mean score achieved a 5‐point improvement in the Physical Function domain as compared to their preoperative baseline score.

**Figure 2:**
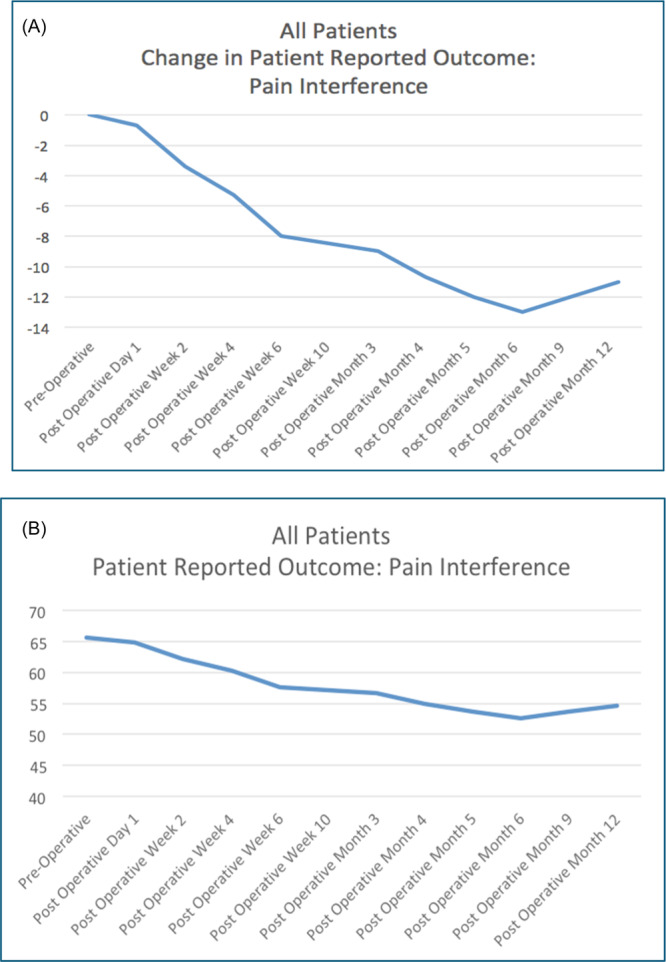
Pain Interference domain *T*‐scores for patients with impending or realized pathologic fracture due to metastatic cancer to bone, before and after orthopedic intervention. (A) Average *T*‐score for the entire cohort over time, where a score of 50 represents historical control of the average US population (standard deviation ±10), and a higher score reflects more pain. (B) Change in the average *T*‐score of the studied cohort relative to their test taken at baseline, immediately before surgery, with a change in score of +5.0 set as the Minimally Clinically Important Difference.

Tables 3 and 5 show the results of the Pain Interference domain of the PROMIS CAT taken by the patients. The average T‐score at the time of their preoperative baseline assessment was 65.6 (standard deviation 8.67, standard error 0.96). For this cohort, 72.8% (*n* = 59) achieved at least a 5‐point improvement in their Pain Interference score following surgical intervention for their impending or realized fracture, while 64.2% (*n* = 52) achieved a 7.5‐point improvement. The average time to achieve the 5‐point level of change for the entire cohort was 4 weeks after surgery, while the 7.5‐point change averaged 6 weeks to achieve. As with the Physical Function domain, the improvements noted in the Pain Interference scores were durable, with no substantive regression in Pain scores through the study period, although there was a small uptick in pain scores between the 9‐ and 12‐month periods, increasing from 53.6 to 54.6. A subset of patients, 13.6% (*n* = 11), never achieved any improvement in their Pain Interference score; rather, their scores declined and remained worse than baseline (Figure 2).

**Table 5:** Pain interference domain: T‐scores for patients undergoing orthopedic intervention for impending and realized pathologic fractures due to metastatic cancer to bone, where a score of 50 with a standard deviation of 10 is standardized to the average US population.

Pain interference PRO	All patients *T*‐score mean ± SD (*n*)	Arthroplasty *T*‐score mean ± SD (*n*)	Plate/screw *T*‐score mean ± SD (*n*)	IM rod *T*‐score mean ± SD (*n*)
Preoperative	65.6 ± 8.6 (81)	65.9 ± 7.3 (22)	65.3 ± 9.1 (7)	65.6 ± 9 (46)
Postoperative Day 1	64.9 ± 9.2 (61)	64.3 ± 7.7 (17)	67.3 ± 8.8 (6)	65.8 ± 10.5 (32)
Postoperative Week 2	62.2 ± 7.6 (66)	58.3 ± 9 (20)a,b	62.1 ± 5.1 (7)	64.7 ± 6.6 (34)
Postoperative Week 4	60.3 ± 8.8 (45)^a^	57.3 ± 8.4 (14)	64.9 ± 7.6 (5)	61.3 ± 9.2 (25)
Postoperative Week 6	57.6 ± 8.5 (49)^b^	54.1 ± 8.4 (15)	60.1 ± 4.8 (5)^a^	58.8 ± 8.8 (29)^a^
Postoperative Week 10	57.1 ± 9.0 (45)	54.4 ± 7.9 (15)	62.6 ± 11.9 (4)	58.3 ± 8.9 (25)
Postoperative Month 3	56.6 ± 10.1 (38)	59.6 ± 10.3 (12)	53.5 ± 13.5 (3)^b^	56 ± 9.6 (23)^b^
Postoperative Month 4	54.9 ± 8.1 (35)	57.3 ± 4.3 (10)	53.5 ± 5.9 (3)	54 ± 9.5 (22)
Postoperative Month 5	53.6 ± 8.6 (33)	49.5 ± 8.1 (11)	57.6 ± 1.8 (2)	56.3 ± 7.9 (19)
Postoperative Month 6	52.6 ± 10.9 (37)	49.6 ± 8.5 (13)	54.1 ± 3.3 (3)	55.1 ± 12.4 (20)
Postoperative Month 9	53.6 ± 8.8 (26)	50.6 ± 6.6 (9)	54.1 ± 3.7 (3)	56.3 ± 8.8 (16)
Postoperative Month 12	54.6 ± 8.6 (27)	51.2 ± 6.4 (8)	49.1 ± 15.8 (2)	56.9 ± 8.4 (17)

*Note:* Six patients did not have the reconstruction listed in the REDCap database, but were included in the total cohort, as the remainder of their data was complete. Therefore, the listed numbers of each reconstruction may not add to the number listed in the all patients group.

Abbreviation: SD, standard deviation.

a First time each group's mean score achieved a 5‐point improvement in the Pain Interference domain as compared to their preoperative baseline score.

b First time each group's mean score achieved a 7.5‐point improvement in the Pain Interference domain as compared to their preoperative baseline score.

**Figure 3:**
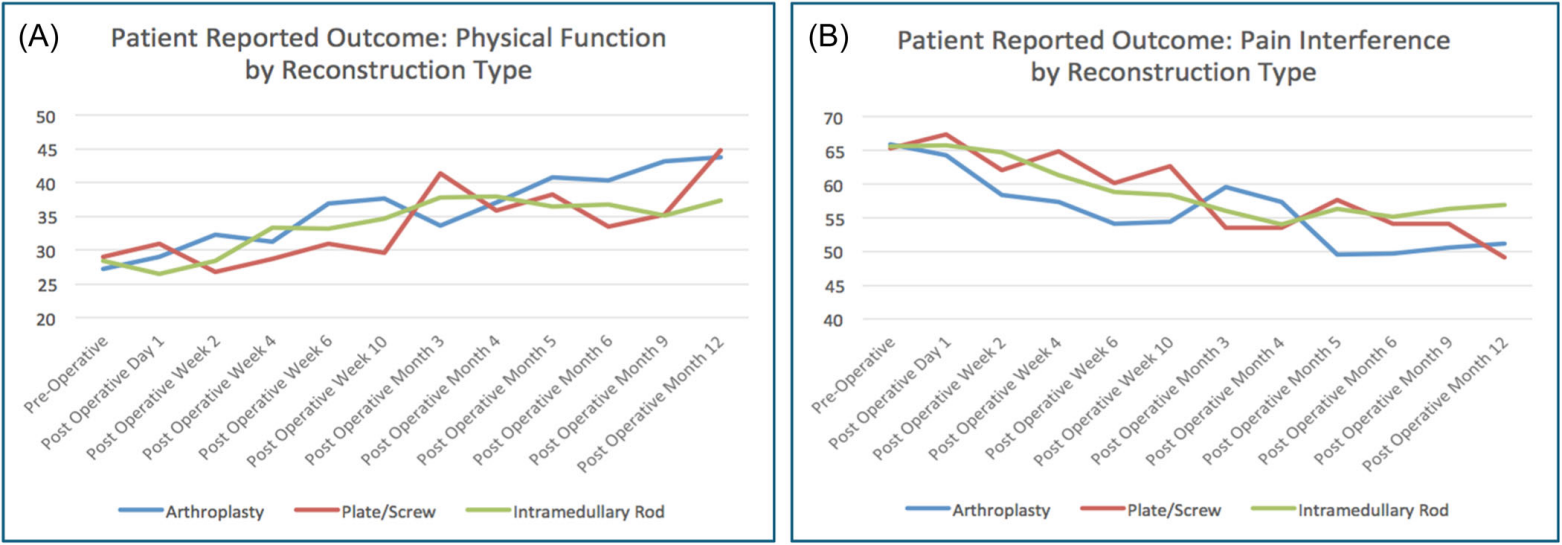
Mean Physical Function and Pain Interference domain *T*‐scores for patients with impending or realized pathologic fracture due to metastatic cancer to bone, before and after orthopedic intervention, separated by reconstruction method. (A) Average Physical Function domain *T*‐score for each reconstruction cohort over time, where a score of 50 represents historical control of the average US population (standard deviation ±10) and a lower score reflects worse function. (B) Average Pain Interference domain *T*‐score for each reconstruction cohort over time, where a score of 50 represents historical control of the average US population (standard deviation ±10) and a higher score reflects worse pain.

Tables 4 and 5 and Figure 3 show the scores of the Physical Function and Pain Interference tests as reported by surgical intervention. The suggestion of the data is that these interventions tend to trend in a similar fashion for the Physical Function and Pain Interference domains, with arthroplasty achieving the 5‐point changes by postoperative Week 2, while the intramedullary rod and plate and screw groups appeared to take longer. However, this splitting of the data into intervention groups was done for understanding the potential homogeneity versus heterogeneity of the data and not for comparison's sake. These groups were not randomized to treatment, so no inference of the superiority of one of these interventions over another can be made, and, therefore, no *p* values were calculated.

**Figure 4:**
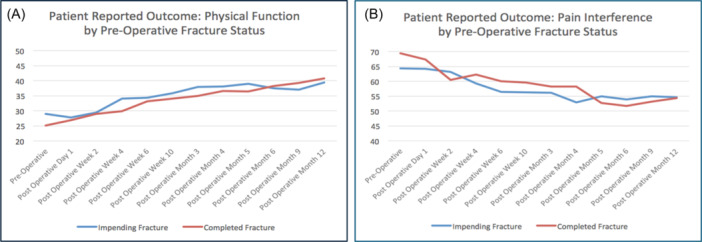
Mean Physical Function and Pain Interference domain *T*‐scores for patients with impending or realized pathologic fracture due to metastatic cancer to bone, before and after orthopedic intervention, separated by the status of preoperative impending or realized fracture. (A) Average *T*‐score for Physical Function for patients with either impending or realized fractures, where a score of 50 represents historical control of the average US population (standard deviation ±10). (B) Average *T*‐score for Pain Interference for patients with either impending or realized fractures, where a score of 50 represents historical control of the average US population (standard deviation ±10).

Figure 4 demonstrates the Physical Function and the Pain Interference scores when the cohort was separated into the group that had impending fractures versus completed fractures. The Completed Fracture group had worse Physical Function (*T*‐score 25.1) and Pain Interference (*T*‐score 69.4) scores at the preoperative baseline assessment than the Impending Fracture group (28.9 and 64.4, respectively), but both groups trended together throughout the postoperative period, with nearly identical scores at the 12‐month mark. Again, statistical differences were not determined between the groups, as the study was established to investigate the response of the cohort as an entire group, and this secondary splitting into groups was to investigate potential sources of heterogeneity or confounding factors within the group, in an effort to generate questions for future study.

## 4 Discussion

Our results suggest that intervention with orthopedic surgery for impending or realized pathologic fractures due to metastatic cancer to bone leads to a relatively rapid improvement in pain and function. As a group, the 5‐point improvement in the Physical Function domain of the PROMIS CAT was observed during the sixth week postoperative assessment, while the Pain Interference domain demonstrated a 5‐point improvement by the fourth week after surgery.

Overall, our findings demonstrate that this cohort of patients has a very low level of preoperative function and a very high level of pain. The Physical Function domain scores were more than 2 standard deviations below the functional score of the average within the US population (*T*‐score 27.8 vs. 50, respectively). Likewise, the Pain Interference score placed the average patient in this study 1.5 standard deviations above the US population (*T*‐score 65.6 vs. 50, respectively). This underscores the predicament this group of patients must face: undergoing a relatively invasive surgery when their health and well‐being are declining. By the end of the year after surgery, this group improved in Physical Function PROMIS score by 12 points on the T‐score, from 27.8 preoperatively to 39.8 at the end of the year. While this difference more than doubles the presumed MCID, the group still remained 1 standard deviation below the US population average. Likewise, Pain Interference T‐scores improved a full standard deviation, from the preoperative baseline of 65.6 to the 1‐year follow‐up value of 54.6. This places the pain scores within 0.5 standard deviation of the mean value of the US population

It is important to note that not all patients improved after surgery. While 63.0% of patients achieved a 5‐point improvement in the Physical Function Domain and 72.8% achieved the 5‐point MCID improvement in Pain Interference, the remainder did not see a MCID in function or pain scores after surgery. In fact, 18.5% (*n* = 15) had worse Physical Function domain scores, and 13.6% (*n* = 11) had worse Pain Interference domain scores following surgical intervention. For this subset of patients, scores worsened after surgery and never recovered on subsequent evaluations. Follow‐up for this subset of patients was short, ranging from 2 to 10 weeks, with an average of 3.7. Of the 15 with worsened self‐reported function, 4 of these patients died during this short time, while another 6 reported ill‐health as the reason to drop out of the study at these time‐points; the remaining 5 were lost to follow‐up. This underscores the need to fully assess patient needs and goals in relation to their prognosis and how difficult these evaluations and decisions can be.

The data demonstrated several nuances that are worthy of discussion. First, while acknowledging that this study did not compare subgroups, this data did not suggest a difference in the tested PROMIS score outcomes for patients with a completed fracture versus an impending fracture. The medical literature has reported that prophylactic fixation of an impending fracture has many advantages over treating a completed pathologic fracture, including decreased operative invasiveness, greater likelihood of discharge to home rather than skilled nursing facilities, and better postoperative function [2, 4, 5, 8]. For example, Ward et al. reported on their experience with 97 impending pathologic fractures and 85 completed fractures for metastases to the femur, and they found that the impending fracture group had shorter hospital stays and a greater likelihood of resuming “support‐free ambulation” than the completed fracture group [9]. Our data does not necessarily contradict these observations, but does reflect that both groups tend to improve at similar rates and achieve similar PROMIS scores postoperatively. While the data from this study show that the completed fracture subgroup seemed to have lower preoperative functional scores (*T*‐score = 25.1) than the impending subgroup (*T*‐score = 29), these scores equalized by postoperative week 2 (*T*‐score = 28.9 and 29.4, respectively), and then improved with a similar trend over the course of the year (Figure 4A). Likewise, the Pain Interference domain demonstrated a worse initial score for the completed fracture group (*T*‐score = 69.4) versus the impending cohort (*T*‐score = 64.4), but the difference diminished by the second week following surgery (*T*‐score = 60.5 and 63.1, respectively), and trended together for the rest of the year. This does not mean that we would be unconcerned if an impending fracture were to progress to a completed fracture, nor would we recommend forgoing prophylactic fixation simply on a wait‐and‐see basis. Realized fractures have consequences that were not investigated in this study, including comorbidities that would potentially prevent surgery. Rather, this study suggests that, for those with impending or completed fractures due to metastases who are deemed appropriate for surgical intervention, both groups may reasonably expect steady and similar postoperative improvement in self‐reported pain and function scores.

The second nuance worthy of discussion involves the analysis of outcomes based on orthopedic implant selection. While the type of implant was not randomized, and therefore no inference is suggested that one implant is “better” than another, our expectation was that the intramedullary nail group would recover faster and more consistently than the arthroplasty group. Our clinical assumption was that an intramedullary nail is less invasive than an arthroplasty surgery; therefore, we predicted that the arthroplasty group would lag behind the intramedullary group in recovery, as reflected by the self‐reported postoperative pain and function scores. In fact, we observed the opposite. The arthroplasty group achieved a 5‐point improvement in the Physical Function domain by the second week after surgery, while the same 5‐point improvement was not seen in the intramedullary nail group until Week 4 and not until the third month after surgery for the plate and screw group. Similarly, the arthroplasty group saw both a 5 and a 7.5 point improvement in pain scores by the second week postoperative visit, while the intramedullary group and plate and screw group did not demonstrate a 5‐point improvement until the sixth week after surgery; these two groups did not achieve a 7.5 point improvement in pain until the 3 months visit after surgery. These data would suggest that, when indicated, arthroplasty is well tolerated by this patient population and can lead to rapid improvement in self‐reported outcomes for both physical function and pain.

There are several limitations to this study. The first limitation centers on the design of the study. The study allowed and included a substantial amount of heterogeneity within the confines of the investigated population. That is, while the population was limited to patients with metastatic disease to bone who were treated with orthopedic surgery, the study included substantial heterogeneity, including a wide range of patient ages (33−92 years); patients treated with various neoadjuvant regimens; various orthopedic implants; multiple anatomic sites; and both impending and completed fractures. We allowed the variability to assess this population as a whole. The population of patients is, by definition, a heterogeneous group. Narrowing our inclusion criteria would have helped answer a group of more specific questions, but would have limited the general nature of the original question of the study. Specifically, we wished to determine how long it takes a patient to realize functional and pain‐related improvement after orthopedic surgery for metastatic disease to bone.

Another issue with the design of the study is that this was not a comparison study. We did not set out to compare differences in outcomes between the various types of orthopedic implants for the treatment of metastatic disease to bone. It is tempting to look at the data and search for clues as to which implant did better than another. However, each implant was selected by an orthopedic oncologist specifically to address an individual issue within an individual patient. In this way, the most these data can infer is that, “when an orthopedic oncologist deems this implant appropriate, here are the outcomes observed.” Another limitation of the study is the lack of completeness of the data collected. Nineteen patients that were consented had to be excluded due to a lack of data entered into the REDCap system. Likewise, 18 patients did not complete the surveys by the end of the year despite being listed as alive and healthy enough to participate. A substantial number of patients (35.8%, *n* = 29) only completed 3−5 surveys. This missing data weakens the overall robustness of the results. That said, the study was undertaken in a prospective fashion, and considerable effort was made to follow patients during a time in their lives when they have immense stress and health‐related concerns.

These results should inform orthopedic oncologists and patients about the potential postoperative course in this group of patients facing difficult decisions regarding the benefits of surgical interventions during a time when the course of the patient's cancer is likely progressing. Future studies should narrow the anatomic sites for specific studies and compare the various orthopedic implants in a prospective randomized fashion.

## 5 Conclusion

Patients with impending or completed pathologic fractures due to metastatic cancer can see clinically important improvements in pain and function, as measured by PROMIS CAT Pain Interference domain and Physical Function domain testing, in an average of 4 and 6 weeks, respectively.

## Disclosure

The authors have nothing to report.

## Synopsis

Orthopedic surgery for impending or completed pathologic fractures in patients with metastatic cancer can result in clinically important improvements in pain and physical function in 4−6 weeks, respectively, based on patient‐reported outcomes.

## Data Availability

Data is available on request due to privacy/ethical restrictions.
